# A scalable blockchain-enabled federated learning architecture for edge computing

**DOI:** 10.1371/journal.pone.0308991

**Published:** 2024-08-16

**Authors:** Shuyang Ren, Eunsam Kim, Choonhwa Lee

**Affiliations:** 1 School of Data Science and Artificial Intelligence, Wenzhou University of Technology, Wenzhou, China; 2 Department of Computer Engineering, Hongik University, Seoul, Korea; 3 Department of Computer Science, Hanyang University, Seoul, Korea; Jazan University, SAUDI ARABIA

## Abstract

Various deep learning techniques, including blockchain-based approaches, have been explored to unlock the potential of edge data processing and resultant intelligence. However, existing studies often overlook the resource requirements of blockchain consensus processing in typical Internet of Things (IoT) edge network settings. This paper presents our FLCoin approach. Specifically, we propose a novel committee-based method for consensus processing in which committee members are elected via the FL process. Additionally, we employed a two-layer blockchain architecture for federated learning (FL) processing to facilitate the seamless integration of blockchain and FL techniques. Our analysis reveals that the communication overhead remains stable as the network size increases, ensuring the scalability of our blockchain-based FL system. To assess the performance of the proposed method, experiments were conducted using the MNIST dataset to train a standard five-layer CNN model. Our evaluation demonstrated the efficiency of FLCoin. With an increasing number of nodes participating in the model training, the consensus latency remained below 3 s, resulting in a low total training time. Notably, compared with a blockchain-based FL system utilizing PBFT as the consensus protocol, our approach achieved a 90% improvement in communication overhead and a 35% reduction in training time cost. Our approach ensures an efficient and scalable solution, enabling the integration of blockchain and FL into IoT edge networks. The proposed architecture provides a solid foundation for building intelligent IoT services.

## Introduction

The rapid development of the Internet of Things (IoT) industry has led to the emergence of various intelligent IoT services, including smart homes, smart transportation, and smart healthcare [1]. To meet the demands of these scenarios, the extensive deployment of IoT devices is necessary, resulting in a vast volume of generated IoT data. According to recent reports, the data generated by IoT devices are projected to reach a staggering 73.1 ZB by 2025 [2]. Although the abundance of IoT data presents opportunities for data-driven machine learning, it also introduces new challenges. Machine learning requires large and diverse datasets to build accurate and robust models that can be satisfied using IoT data [3]. However, machine learning nodes are typically deployed in the cloud, and transporting large amounts of data from data sources to the cloud can significantly strain the network bandwidth. Moreover, IoT data often contains sensitive information, necessitating the implementation of privacy protection measures during data transmission to prevent issues such as data leakage and tampering [4].

Recently, a groundbreaking machine learning scheme known as federated learning (FL) has emerged, offering solutions to the challenges of data transmission and privacy in the context of machine learning [5]. FL nodes tap into their local data for model training and upload the resultant model parameter updates to a central aggregation server that computes the new global model. Subsequently, the learning nodes continue to start a new training cycle by downloading the newly released global model. FL components can be distributed across edge regions, and model training can be performed independently without exposing the raw data, which enhances system scalability and data privacy. However, without an appropriate malicious detection scheme, FL is susceptible to poisoning attacks. Moreover, the centralized aggregator is likely to become a performance bottleneck, resulting in potential inefficiencies [6].

Blockchain, the underlying technology of cryptocurrencies, possesses the characteristics of traceability, tamper-proof, and decentralization [7]. Since the launch of Bitcoin, blockchain technology has gained significant attention in academic research and has evolved into various variants to meet the requirements of diverse real-world scenarios [8]. Its decentralized nature aligns well with distributed FL, making blockchain technology a potential solution for addressing the challenges faced by FL. The traceability and tamper-proof features of blockchain contribute to enhancing the security of FL, whereas its decentralized nature mitigates the risk of single-point failures in FL processing setups. Therefore, integrating blockchain and FL opens new possibilities and unveils a promising avenue for improving performance and security [9].

In early trials, Bao et al., Majeed et al., and Nguyen et al. proposed federated learning blockchain (FLChain) frameworks [10–12]. These frameworks enable nodes to share locally trained model updates through the blockchain, thereby enhancing the security of the model training process. Furthermore, based on the blockchain, the global model is aggregated in a peer-to-peer manner instead of a centralized aggregator, thus solving the single-point-of-failure issue and increasing system availability. However, the FLChain framework still faces some challenges. First, both blockchain and FL are resource-consuming systems; thus, implementing both systems in resource-limited edge computing environments is not straightforward. Second, many existing studies utilize Proof of Work (PoW) or Practical Byzantine Fault Tolerance (PBFT) protocols to maintain the blockchain, leading to inefficiencies and increased latency within the system. Recently, Cao et al. and Ko et al. proposed new approaches to FL using a Direct Acyclic Graph (DAG)-based blockchain [13, 14]. These approaches avoid the use of traditional resource-hungry consensus protocols. However, the nonlinear topology of the DAG chains likely causes a low convergence rate of the target model in FL, which negatively affects the training efficiency.

This paper presents our FLCoin architecture for integrating blockchain and FL into edge environments. From the perspective of blockchain, we exploit a committee-based optimized Byzantine Fault Tolerance (BFT) consensus protocol to improve efficiency and scalability. The scalability of the system was significantly improved by implementing the consensus protocol within the limited scope of the consensus committee. We take advantage of the FL process to determine the consensus committee, instead of using resource-intensive approaches, such as PoW or voting schemes. Committees are formed dynamically based on the model updates processed by the nodes during FL operation. In addition, we adopted a linear communication approach to replace the all-to-all communication mode used by PBFT, further enhancing the system efficiency. From the FL perspective, local model updates and aggregated global models are recorded in the form of updates and model blocks on blockchain to ensure security. In terms of node management, we introduced an identity chain to enhance control over node permissions. Overall, our FLCoin architecture offers a harmonious solution for integrating blockchain and federated learning with high scalability and efficiency in edge computing environments. The main contributions of this study are as follows:

- We propose FLCoin, a harmoniously integrated architecture of blockchain and federated learning for edge computing environments.
- We design a committee-based BFT consensus protocol that ensures high scalability. The consensus committee is dynamically formed through the federated learning process.
- To enhance system efficiency, we further optimize the BFT consensus protocol, reducing the communication cost during the consensus operation.
- We optimize blockchain storage by releasing obsolete historical, significantly reducing the system’s consumption of storage space.

The remainder of this paper is organized as follows. In the “Background” section, we briefly discuss the technical background for our study. The “System overview” section then introduces the overall architectural design of the FLCoin protocol and the protocol is described in further detail in the following section of the “Protocol operation”. Subsequently, we present an analysis of the consensus protocol security and report our performance evaluation results in the “Theoretical analysis” and “Evaluation” sections. We then summarize previous relevant studies in the “Related work” section. After discussing the limitations of our current study and our future work in the “Limitations and future work” section, we conclude this paper in the “Conclusion” section.

## Background

In this section, we present a concise technical background of the topics explored in this study. First, we cover blockchain technology and highlight several consensus protocols commonly used in blockchains. Then, we provide an overview of federated learning (FL), a distributed machine learning approach that ensures data privacy by avoiding the need to share local data. Finally, we introduce FLChain, a general federated learning-blockchain integration framework.

### Blockchain and consensus protocols

Blockchain is a distributed and shared ledger that operates on a peer-to-peer network. It uses blocks as storage units containing transaction information, along with the hash value of the previous block, to form a chain storage structure. Participating nodes in the blockchain equally verify and store the data through consensus protocol operations, thereby maintaining system consistency. This decentralized structure provides immutability, reliability, and security to blockchain systems.

Blockchains can be classified into two categories: permissionless and permissioned. In permissionless blockchains, nodes can participate in transaction verification and block creation without restrictions. To append a new block, Proof of Work (PoW), which is a computing-consuming hash puzzle, is widely adopted by permissionless blockchain networks, such as Bitcoin and Ethereum [7, 15]. In contrast to permissionless blockchains, permissioned blockchains mandate node registration and identity authentication for participation in the block creation process. To achieve consensus in the network, Practical Byzantine Fault Tolerance (PBFT), a communication-based consensus protocol, is applied to permissioned blockchains, such as the Hyperledger Fabric blockchain [16, 17]. Unfortunately, neither PoW-based nor PBFT-based blockchains are well-suited for the integrated architecture of blockchain and FL. Operating PoW requires vast computing resources, and the long consensus latency of PoW unquestionably decreases the performance of blockchain-based federated learning systems, such as delaying the global model aggregation process. However, despite the fact that PBFT is a computing resource-saving and efficient scheme compared with PoW, it imposes a significant communication burden on blockchain-based FL nodes owing to its demand for large bandwidth resources, resulting in scalability issues and suboptimal performance.

A hybrid consensus protocol is a promising scheme that combines the advantages of permissionless and permissioned consensus protocols to enhance efficiency and scalability and overcome the limitations of traditional blockchain consensus protocols [18]. Kogias et al. proposed Byzcoin, which combines PoW and PBFT, where PoW acts as a consensus membership proof mechanism for dynamically constructing consensus committees to execute the PBFT consensus protocol [19]. Committee-based consensus processing improves the scalability of the system while lowering communication costs. However, the computing resource consumption caused by the introduction of the PoW protocol for consensus committee election remains a concern.

### Federated learning

Federated learning (FL), also known as collaborative learning, is a novel machine learning framework proposed in 2016 [5]. The fundamental concept of FL is to jointly train a model without data exposure, where nodes locally train their dataset and share model parameter updates for global model aggregation instead of sharing raw learning data. In classic FL, models are trained in a distributed manner across nodes in edge computing environments, and the training process is coordinated by a central server. The model training process can be divided into several steps: First, the central node distributes an initialized model to all participating nodes to start the model training; Second, the training nodes separately train their own dataset and upload the model parameter updates to the central node; Subsequently, based on the received updates, the central node aggregates a new global model; Finally, the central aggregator sends the latest global model to the training nodes to start another round of model training. This process is iterated until the obtained global model satisfies the training task requirements, such as the desired accuracy.

FL proposes a solution for enhanced data privacy and increased scalability. However, FL still faces several challenges. One significant issue is vulnerability to malicious nodes, particularly in edge computing environments where trust cannot always be assured. Owing to the lack of a detective scheme, FL is susceptible to poisoning attacks, where malicious nodes might attempt to disrupt global model aggregation by uploading fake local model updates, thereby delaying the learning operation. Another challenge stems from the heavy reliance on the central node responsible for aggregating the global model. This centralization could result in a single point of failure, affecting system availability and overall performance. Furthermore, the communication overhead on the central aggregator imposes limitations on scalability, hindering the ability of the system to handle a larger number of nodes efficiently.

### Blockchain-based federated learning

In this paper, we refer to a set of blockchain-based FL systems as FLChain. FLChain enhances the security, availability, and scalability of FL by leveraging decentralized and tamper-proof blockchain technology. The correctness and tamper-proof nature of the updates can be ensured by adding updates from the training nodes to immutable blocks after verification, thereby improving the training reliability. Moreover, the adoption of blockchain eliminates the need for a central node in the global model aggregation, further enhancing the availability and scalability of FL. These advantages have led to the emergence of FL-blockchain integration as a new research trend. Building on [10, 11], Nguyen et al. [12] proposed a general FLChain framework that integrated blockchain into FL.

In a blockchain-based FL system, the model training process is executed in a decentralized manner. First, the training nodes download the initialized model from the blockchain and start local training independently using their own datasets. Second, the training nodes upload the local model updates in the form of blockchain transactions. The miner nodes of the blockchain then verify the transactions, pack them into blocks, and determine a new appended block using a consensus protocol. An edge node can simultaneously act as a training and mining node, or it can participate in model training or blockchain maintenance operations. Subsequently, the training nodes download a new block containing all the updates, aggregate the latest global model, and start the next round of model training. This process is repeated until the trained model achieves the required accuracy.

Although FLChain exhibits considerable potential for enhancing classic FL, there are concerns regarding the associated costs of adopting blockchain technology. Most existing FLChain architectures rely on classic blockchain consensus protocols such as PoW and PBFT. However, the use of PoW consumes substantial computing resources and introduces high latency, while the communication complexity of the PBFT protocol hinders scalability. Furthermore, the network-wide verification of all model updates uploaded by the nodes consumes additional resources. In addition, as the number of participating nodes increases, the size of the block containing the model updates also increases, placing a significant burden on the system for broadcasting the block. To this end, this study proposes a new integrated architecture of blockchain and FL to address the limitations of the existing FLChain frameworks.

## System overview

This section presents the system model and assumptions as well as an overview of the FLCoin framework. The notations used in the paper are shown in Table 1.

**Table 1:** Main notations used in the paper.

Notation	Description
*N*	Edge nodes cluster in the system
*PK*_*i*_	The public key of edge node *i*
*SK*_*i*_	The private key of edge node *i*
*s*	The fixed number of update shares in the sliding window
*j*	Model training task
*L*	The total number of iterations of global model aggregation
*K*	The pre-defined number of updates needed for each iteration of global model aggregation
K	A cluster formed by the nodes participating in a specific model training iteration
*M*_*j*,*l*_	The global model block for the *l*_*th*_ iteration for task *j*
*U*_{*j*,*l*},*k*_	The local model update block uploaded by node *k* based on *M*_*j*,*l*−1_, k∈K
*ω*_*G*_	The global model parameters
*ω*_*k*_	The local model update trained by node *k*, k∈K
*D*_*k*_	The data set used by node *k* for computing local update, k∈K
*C*_*k*_	The contribution of node *k*, k∈K
*T*_*train*,*k*_	Local computation time of node *k*, k∈K
C	Consensus committee

### System model and assumption

Control of the authority of the system participants is achieved through a permissioned blockchain, where each participating node possesses unique key pairs (*PK*_*i*_, *SK*_*i*_) for authentication. In addition, for efficient node management, we utilize an identity chain that records the identity information of each node. The identity chain is maintained by a predefined group of nodes, known as managers, which collect the identity information of the participating nodes for system operation, and generate identity blocks. The generation rate of the identity blocks depends on the system *epoch*, which is a fixed period, such as a day or week. During the operation of epoch *e*, nodes that intend to participate in epoch *e* + 1 submit their information to the managers. At the end of epoch *e*, the managers generate a new identity block of epoch *e* + 1 for the system nodes. This dynamic process allows nodes to join or leave the system as required, thereby ensuring flexibility and adaptability within the network.

The system leverages a standard, eventually synchronous communication model to ensure both safety and liveness; that is, the system can operate asynchronously for a defined duration, switch to a synchronous state, and maintain it for a specified time [20]. We adopted a Byzantine failure model in which malicious nodes could delay communication or forge information. However, these nodes cannot indefinitely delay messages from honest nodes or break the digital signature cryptography. Furthermore, while we permit malicious nodes to possess system resources, we enforce a resource threshold that ensures that the total resources held by the malicious nodes remain below a specified limit, which is set at 25% for safety concerns.

### FLCoin architecture framework

**Fig 1:**
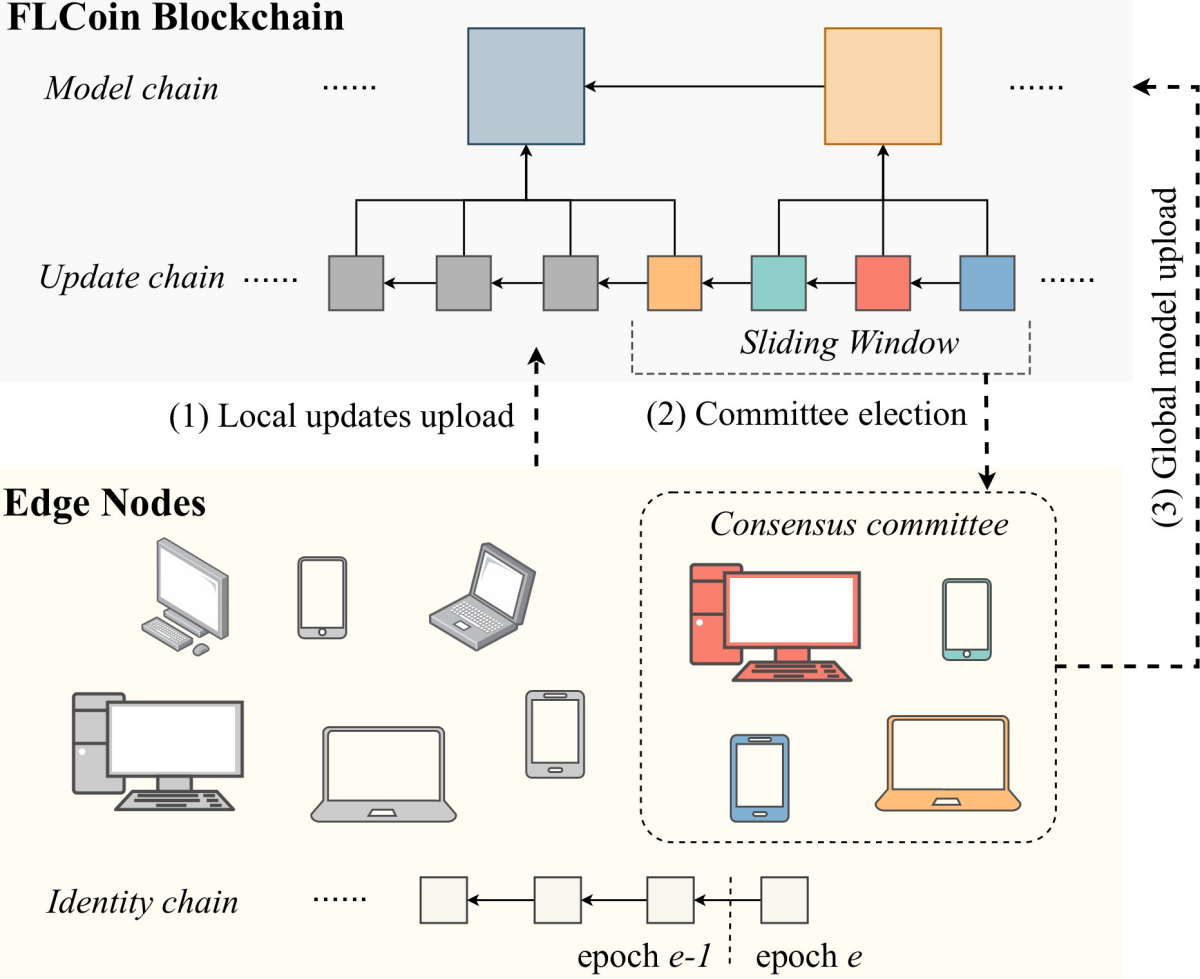
Overall architecture of FLCoin.

We propose FLCoin, as illustrated in Fig 1, to solve the issues faced by the existing integrated architectures of blockchain and FL. The architecture comprised two block types: update and model blocks. The update blocks contain the local model update information submitted by the edge nodes, and the model blocks include the parameters of the global model aggregated from the updates. To ensure the integrity and immutability of model updates, an update block must reference both its previous update block and the model block that holds the global model parameters required for the current training iteration. Additionally, to enhance the scalability of the system while minimizing the resource cost through blockchain consensus operation, we adopted a committee-based BFT consensus protocol. Furthermore, we optimize the protocol operation to reduce the network resources required for the consensus protocol.

In the FLCoin architecture, the edge nodes participate in FL processing and submit update blocks to earn *shares*, which serve as proof of their membership in the consensus committee. The committee was established using a fixed-length sliding window, and the members changed dynamically as the sliding window moved forward, with new update blocks being added. The nodes that no longer hold membership shares in the current window lose their eligibility to participate in the consensus process. The consensus committee was responsible for running the consensus protocol and verifying the update blocks. To ensure robust validation, a two-step verification process was adopted. The first step involved honest-training checking, in which the training dataset size and local training time were compared. The second step was accuracy checking, which verified the accuracy using the dataset of each committee member as the test dataset. During the FL process, the contribution values of the nodes are calculated based on the training dataset sizes. The contribution values are further utilized by the consensus committee members to divide the rewards, and the node with the highest contribution value becomes the committee leader.

Under the leadership of the current leader, the consensus committee executes the BFT protocol and generates a block containing the aggregated parameters of the global model. To improve the efficiency of the protocol operation, an optimistic fast protocol is introduced, which accelerates the consensus process when all the committee nodes are honest. In addition, a backup protocol is used to ensure the safety and liveness of the consensus protocol in the presence of malicious nodes. To further optimize the resource consumption, the all-to-all communications of the consensus protocol were replaced with linear communication patterns, thereby reducing the overall communication resource consumption.

## Protocol operation

In this section, we discuss the protocol operations composed of two steps. The first step is the FL-based consensus committee election and the second step is the consensus protocol operation, which includes fast and backup protocols.

### Federated learning-based consensus committee election

In this section, we describe the operation of our federated learning-based consensus committee election.

#### Federated learning in FLCoin

**Fig 2:**
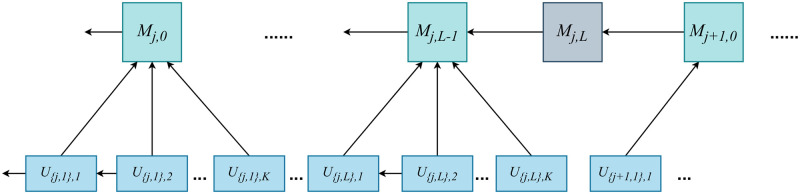
Blockchain structure of FLCoin.

In FLCoin, the FL operation that obtains the final global model with the desired accuracy for the learning tasks is recorded in a two-layer blockchain as illustrated in Fig 2. The learning process is executed using edge nodes cluster *N* = {1, 2, …*n*}. For a specific learning task *j*, *L* iterations of the global model aggregation are required to achieve the desired accuracy. In each iteration, a pre-defined total number of local model updates, denoted by *K*, were performed. The *l*_*th*_ aggregated global model is stored in the model block *M*_*j*,*l*_. Based on the *l*_*th*_ global model, the edge node *k* trains its local dataset and generates a local model update block *U*_{*j*,*l*+1},*k*_. To prevent malicious behavior of *lazy node*, where the nodes repeatedly send identical local model updates, we allow each node to upload only one update block based on the same global model. During the learning process, *M*_*j*,0_ represents the initialization of the learning task and *M*_*j*,*l*_, which contains the final global model parameters, is the last model block for learning task *j*, which indicates the termination of the training process. Therefore, *U*_{*j*,1},0_ is the first update block of learning task *j* and *U*_{*j*,*L*},*K*_ serves as the last update block.

To update the *l*_*th*_ global model of task *j*, total *K* local model updates are required. Correspondingly, the nodes generate update blocks based on *M*_*j*,*l*_ to construct a subset of nodes K and |K|=K. Specifically, the edge node k∈K utilizes its local dataset *D*_*k*_ to compute a local model update based on the global model in block *M*_*j*,*l*_. The FL objective is to obtain the optimal global model parameters *ω*_*G*_ that minimize the loss function *F*(*ω*) across all datasets $D_{K}=\cup_{k\in K}D_{k}$. A data sample *d*_*i*_ ∈ *D*_*k*_ can be represented as *d*_*i*_ = {*x*_*i*_, *y*_*i*_}, where *x*_*i*_ is a *d*-dimensional volume vector, and *y*_*i*_ is a scalar value. Consequently, the loss function can be defined as follows:

$$\begin{array}{c} F\left(\omega \right)=\frac{1}{|D_{K}|}\sum_{k=1}^{K}\sum_{d_{i}\in D_{k}}\frac{{\left(x_{i}^{⊤}\omega -y_{i}\right)}^{2}}{2} \end{array}$$ (1)

Then, the local model update $\left(\omega_{k},{\left\{\nabla F_{i}\left(\omega_{G}\right)\right\}}_{d_{i}\in D_{k}}\right)$ is computed by using the stochastic variance reduced gradient (SVRG) algorithm. Additionally, the global model update (*ω*_*G*_, ∇*F*(*ω*_*G*_)) is generated using the distributed approximate Newton-type (DANE) method.

#### Blocks in FLCoin

**Fig 3:**
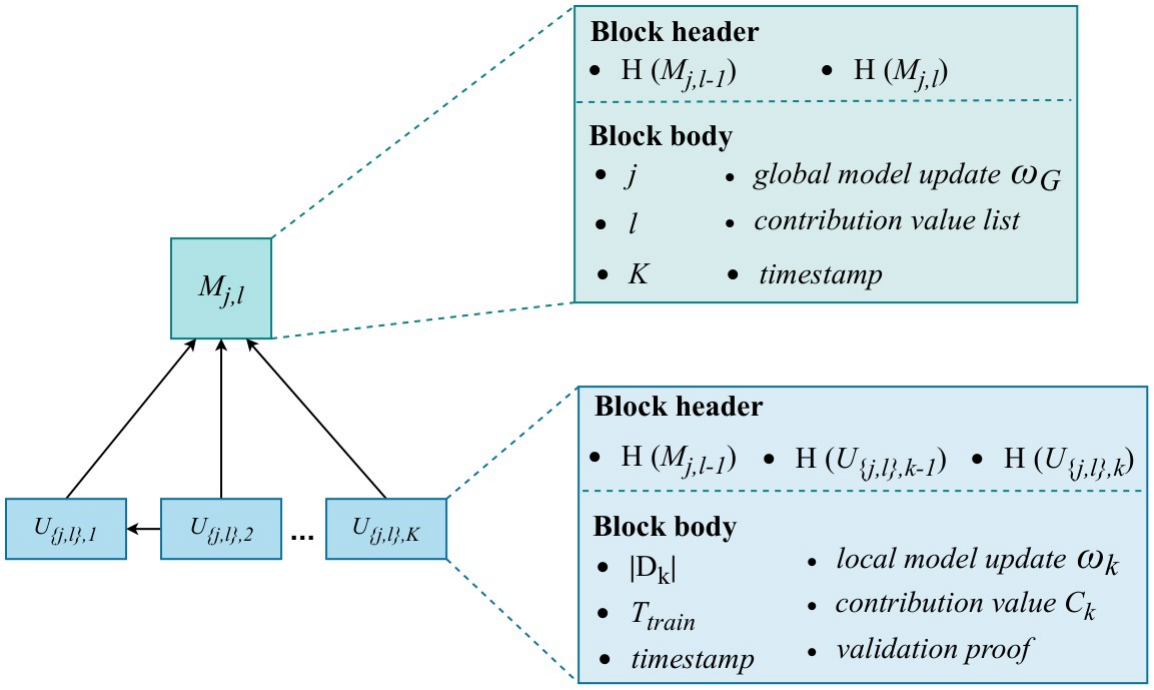
Block contents in FLCoin.

As illustrated in Fig 3, FLCoin system comprises two types of blocks: model blocks and update blocks, each with a header and a body. A model block *M*_*j*,*l*_ header contains two components: *H*(*M*_*j*,*l*−1_) is a pointer to the previous block and *H*(*M*_*j*,*l*_) is the hash value of the current block. The block body contains the learning task number *j*, current iteration number *l*, timestamp, and parameter *K*, which indicate the number of local updates required for global model aggregation. Furthermore, the block body includes the global model parameters and contribution values of all *K* local model updates. For each task, only the final global model satisfied the accuracy requirements. Therefore, to optimize blockchain storage, other model blocks are pruned after obtaining the final model block *M*_*j*,*l*_ for task *j*. To ensure the immutability of the final global model for previous tasks, *M*_*j*,*l*_ contains the hash value of the final model block of the previous task *j* − 1 in addition to the inherent contents in the block header. Moreover, the final model block includes the contribution values of all nodes participating in task *j* training. For node *k*, which generates update block *U*_{*j*,*l*},*k*_, its contribution value *C*_*k*_ is calculated as follows:

$$\begin{array}{c} C_{k}=\alpha \times \left|D_{k}\right| \end{array}$$ (2)

where *α* is a predefined coefficient in the range of (0, 1), and |*D*_*k*_| is included in the block body of *U*_{*j*, *l*}, *k*_, representing the sample size of the data used by node *k* to compute its local model update. Additionally, for the update block *U*_{*j*,*l*},*k*_, the block header contains three components: the hash value of the model block *M*_*j*,*l*−1_ that holds the global model used in the current local training, the hash value of the previous update block *U*_{*j*,*l*},*k*−1_, and the hash value of the current block *U*_{*j*,*l*},*k*_. The update block body contains the local model updates, local training data sample size |*D*_*k*_|, local computing time *T*_*train*_, contribution value, and timestamp. These contents were utilized for model update verification. After verification, the local model update obtains a validation proof, and node *k* adds this proof to complete the generation of a valid update block.

#### Consensus committee conversion

**Fig 4:**
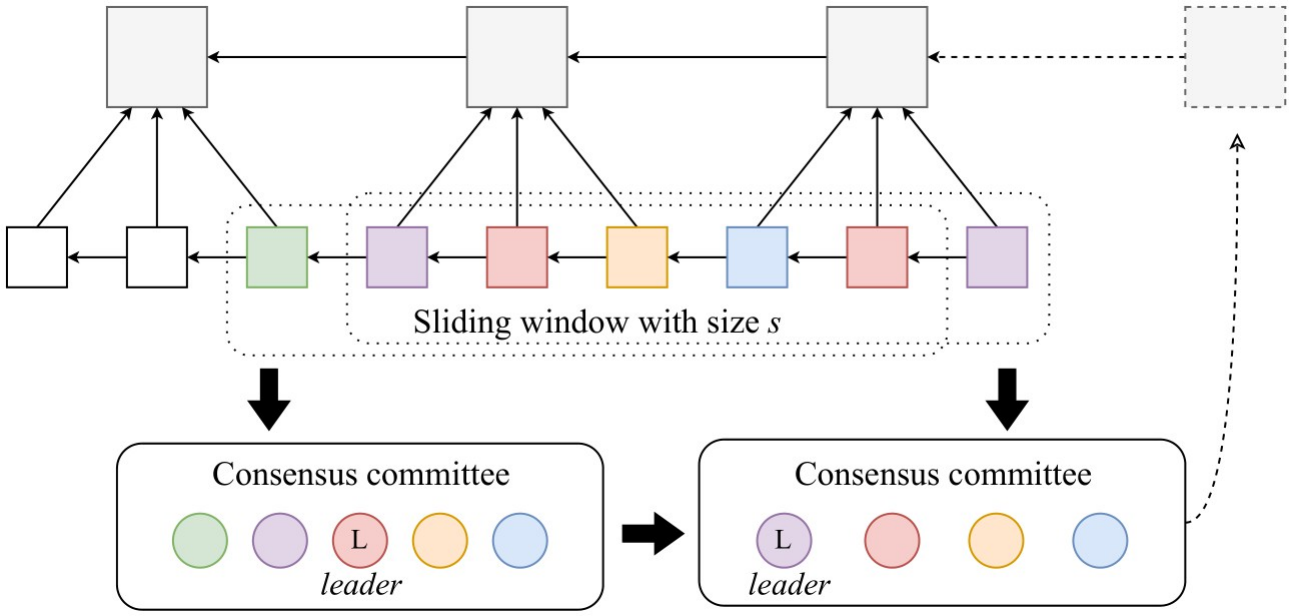
FL-based committee conversion processing.

The global model in FLCoin is aggregated in a decentralized manner, based on blockchain maintained by dynamically formed consensus committees. As illustrated in Fig 4, a committee is constructed based on a sliding window with a fixed size *s* comprising *s* valid update blocks. The committee changes dynamically as the sliding window moves forward when new update blocks are attached. Each update block within the current window represents the membership *share* of the consensus committee. The contribution values of the update block proposers are calculated as the cumulative outcomes of all update shares. The node with the highest contribution value among the proposers was designated as the committee leader. When multiple nodes obtain the same contribution values, the leadership is determined by the timestamps of their first shares. The node that attaches its update block earliest in the current window assumes the role of the committee leader, coordinating the consensus protocol operation. The consensus committee was responsible for validating the newly attached update block and generating a model block. Correspondingly, the rewards include two parts: block validating and block generating; the rewards are distributed based on the contribution value. Therefore, the edge nodes strive to mine additional update blocks to obtain rewards.

To generate an update block, a node *k* trains the latest global model based on its local dataset *D*_*k*_ and then sends its local model update, dataset size |*D*_*k*_|, local training time *T*_*train*_, and its computation resource $R_{k}^{CPU}$ to committee members for validating. A local model update requires a two-step verification to be valid, as denoted by the two procedures in Alg 1. To prevent malicious nodes from deceiving their actual data sample sizes, the first step of verification completes an honest training checking by determining whether Eq 3 holds,

$$\begin{array}{c} T_{train}=\frac{|D_{k}|\times \mu }{R_{k}^{CPU}} \end{array}$$ (3)

where *μ* is the number of CPU cycles required to train one unit of data.

**Algorithm 1 Step 1: Model update verification process**.

 *D*_*k*_: the data set of node *k*

 |*D*_*k*_|: the size of data set *D*_*k*_

 *T*_*train*,*k*_: the local computing time of node *k*

$R_{k}^{CPU}$ : the computation resource of node *k*

 *μ*: CPU cycles needed for training on a unit of data

 *H*: a hash function

 *ω*_*k*_: the local model update of node *k*

 Receiving information from node *k*

1: **Procedure** Honest-training checking

2: **if** $T_{train,k}=\frac{|D_{k}|\times \mu }{R_{k}^{CPU}}$ is correct **then**

3:  Start **Procedure** accuracy checking

4: **else**

5:  Mark node *k* as malicious

6: **end if**

7: **Procedure** Accuracy checking

8: Check the accuracy of *ω*_*k*_ with local data set

9: **if** the accuracy is in the threshold **then**

10:  *hash*_*k*_ = *H*(*ω*_*k*_ || |*D*_*k*_|)

11:  *σ* ← *Sign*(*hash*_*k*_)

12:  Send *σ* to node *k*

13: **else**

14:  Mark node *k* as malicious

15: **end if**

**Fig 5:**
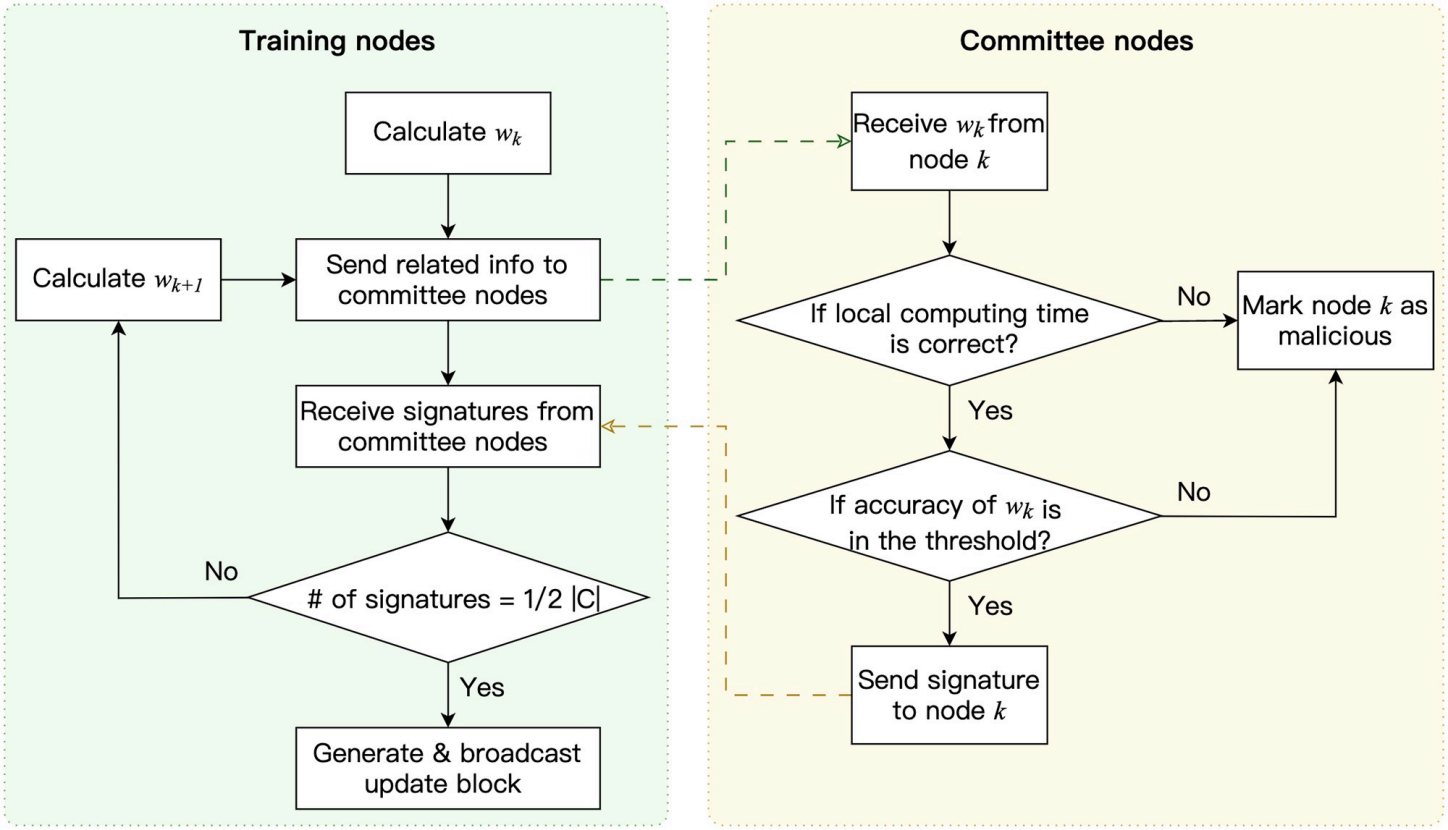
Local model verification and update block generation.

The following operation checks the accuracy of the local model update, which should be within the threshold. Committee members utilized their local datasets as testing datasets to examine the accuracy of receiving local model updates. To update node *k*, if its accuracy is valid, a committee member calculates the hash value as follows:

$$\begin{array}{c} hash_{k}=H(\omega_{k}\;||\;|D_{k}\left|\right) \end{array}$$ (4)

where *ω*_*k*_ denotes the local model update received from node *k*. The committee then signs *hash*_*k*_ with its private key and sends a signed message to node *k*. Notably, consensus is not required during the accuracy-checking operation. When node *k* receives signatures from 2/3 of the committee members, it generates a validation proof, which is a list containing the signatures. Then, node *k* generates an update block and broadcasts it to other edge nodes. When receiving the update block, the edge nodes check the validation proof and add the block to storage. At this point, the valid update block of node *k* is attached to the update chain and node *k* obtains a share of the consensus committee membership. The update block generation operation for edge nodes is presented in Alg 2. The overall operation of local model verification and update block generation is illustrated in Fig 5.

**Algorithm 2 Step 2: Block generating process**

 *D*_*k*_: the data set of node *k*

 |*D*_*k*_|: the size of data set *D*_*k*_

 *d*_*i*_ ∈ *D*_*k*_: the data samples of node *k*,

 *T*_*train*,*k*_: the local computing time of node *k*

C : the Consensus committee.

1: **Procedure** Generating Update block *U*_{*j*,*l*},*k*_

2: **for** all *d*_*i*_ ∈ *D*_*k*_ **do**

3:  Calculate $\left(\omega_{k},{\left\{\nabla F_{i}\left(\omega_{G}\right)\right\}}_{d_{i}\in D_{k}}\right)$

4:  Send |*D*_*k*_| & *T*_*train*,*k*_ & *ω*_*k*_ to C

5:  Receiving signatures $Sigs=\cup_{i\in C}\sigma_{i}$

6:  **if** $\left|Sigs\right|=\frac{2}{3}\left|C\right|$ **then**

7:   Generate validation proof

8:  **end if**

9:  Package update block *U*_{*j*,*l*},*k*_

10: Broadcast *U*_{*j*,*l*},*k*_

11: **end for**

### Operation of the consensus protocol

After sufficient update blocks have been attached to the update chain, the corresponding consensus committee members aggregate the global model in a decentralized manner. The committee then executes a consensus protocol under the coordination of the leader to commit to the newly generated block and append it to the model blockchain. We implemented an optimized BFT consensus protocol that includes fast and backup protocols to enhance the efficiency and scalability of the system. The fast protocol is used to accelerate the consensus processing when the system is in an optimal condition without malicious nodes. However, if the network conditions are not favorable for the fast protocol, we switch to the backup protocol to ensure the security and correctness of the system. Moreover, we utilized a linear communication model that reduced the amount of data exchanged among nodes to minimize the communication cost during the consensus process.

#### Optimistic fast protocol

**Fig 6:**
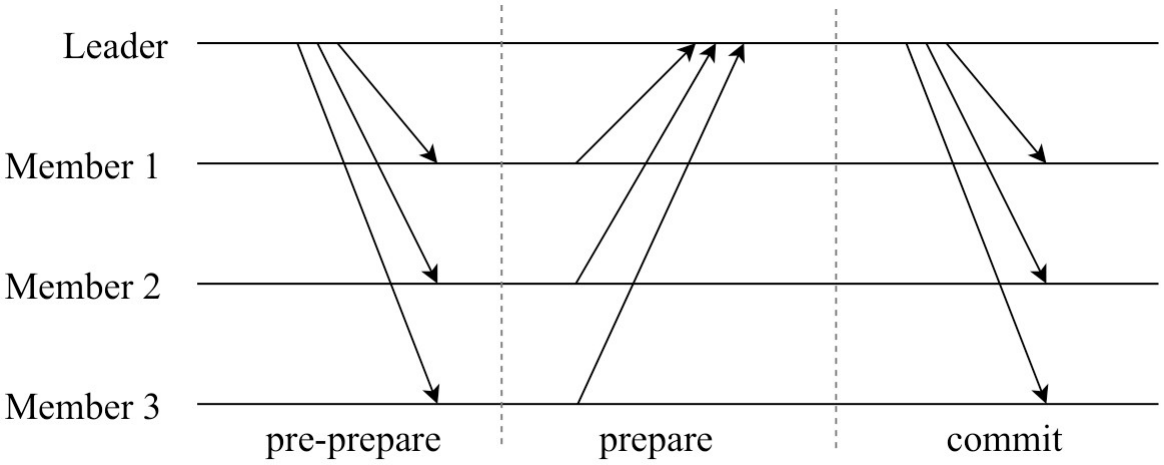
Optimistic fast protocol processing.

Similar to the Zyzzyva and SBFT protocols, our protocol runs an optimistic fast protocol when all *c* nodes in the consensus committee C are honest [21, 22]. The fast protocol comprises three phases: *pre-prepare*, *prepare*, and *commit*, as shown in Fig 6. In the *pre-prepare* phase, the leader broadcasts a “*pre-prepare* message to all consensus committee members, which is expressed as < “*pre* − *prepare*”, *M*_*j*,*l*_, *σ*_*L*_ >. Here, *M*_*j*,*l*_ is the new model block containing the latest global model aggregated from the update blocks $\cup_{k\in K}U_{\{j,l-1\},k}$, and *σ*_*L*_ is the signature of the leader generated with its private key *SK*_*L*_. Upon receiving the message, consensus committee member *i* verifies the contents of the model block and the signature of the leader. If the message is valid, member *i* sends < “*prepare*”, *header*, *σ*_*i*_ > to the leader in the *prepare* phase. In the message, *header* is the block header of *M*_*j*,*l*_ and *σ*_*i*_ is the signature of node *i* generated with its private key *SK*_*i*_. When the leader receives all *“prepare”* messages from *c* consensus committee members, the protocol enters the commit phase. The leader then broadcasts < “*commit*”, *Proof*_*fast*_, *header*, *σ*_*L*_ > to all *c* committee members, where *Proof*_*fast*_ = (*prepare*_1_, …, *prepare*_*c*_). This step confirms the agreement on block *M*_*j*,*l*_, which contains the global model for the subsequent model training.

#### Backup protocol

**Fig 7:**
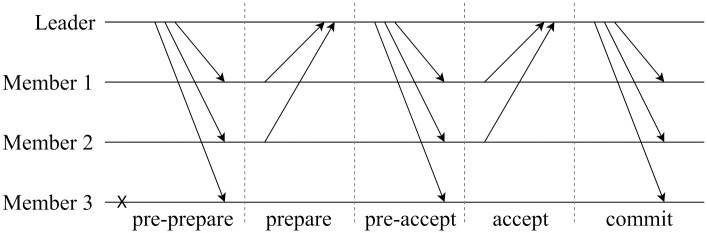
Backup protocol processing.

If the optimistic fast protocol fails to produce results, the system resorts to the backup protocol, which consists of five phases: *pre-prepare*, *prepare*, *pre-accept*, *accept*, and *commit*. The operation of the backup protocol is illustrated in Fig 7. The *pre-prepare* and *prepare* phases of the backup protocol are identical to those of the fast protocol. The leader decides whether to run the fast or backup protocol based on the number of *“prepare”* messages received during the *prepare* phase. If the leader obtains messages from all *c* consensus committee members, the fast protocol is executed, and the system proceeds directly to the *commit* phase. However, if the leader does not receive all messages during the *prepare* phase but obtains more than $\frac{2}{3}c$ messages from the committee members, the backup protocol runs, and the system enters the *pre-accept* phase. During this phase, the leader broadcasts < “*pre* − *accept*”, *Proof*_*back*_, *header*, *σ*_*L*_ > to all members, where *Proof*_*back*_ = (*prepare*_*i*_, …, *prepare*_*j*_), and its length satisfies $\frac{2}{3}c\leq \left|Proof_{back}\right|<c$. When a committee member *i* receives the *pre-accept* message, it sends an *“accept* message < “*accept*”, *header*, *σ*_*i*_ > to the leader during the *accept* phase. The protocol enters the *commit* phase when the leader receives more than $\frac{2}{3}c$ accept messages. Then the leader broadcasts < “*commit*”, *Proof*_*commit*_ *σ*_*L*_ > to all *c* consensus committees, where *Proof*_*commit*_ = (*accept*_*i*_, …, *accept*_*j*_), and its length satisfies $\frac{2}{3}c\leq \left|Proof_{commit}\right|<c$. The system then confirms the agreement on block *M*_{*j*,*l*}_ and starts the subsequent model training processes based on the new aggregated global model contained in *M*_{*j*,*l*}_.

### Epoch transition

In the proposed system, nodes can dynamically join or leave the network, and this process is managed through an identity chain. To facilitate this, system operations were divided into epochs. Both new and existing nodes seeking to join the system in epoch *e* must commit to their identities through the identity chain in epoch *e* − 1. The commitment of identities requires the generation of an identity block at the end of epoch *e* − 1, which includes all the identities of the nodes intended to join epoch *e*. The creation of an identity block is based on an epoch manager group, which is a trusted pre-defined group that assumes responsibility of creating identity blocks and maintaining the identity chain.

### Blockchain storage

**Fig 8:**
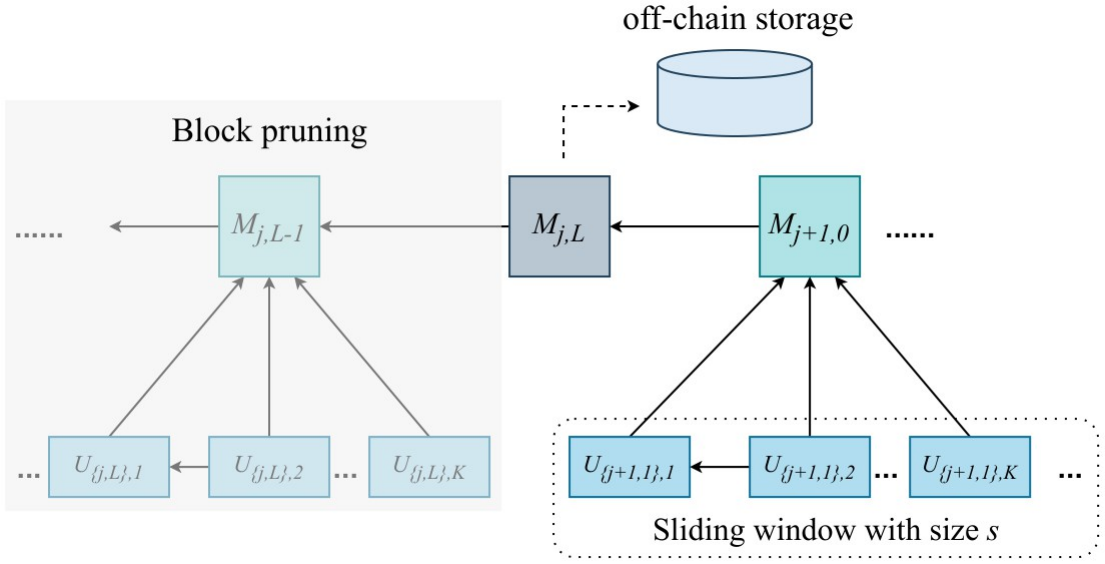
Blockchain storage optimization.

To optimize blockchain storage and reduce the storage costs for nodes, we implemented a *block pruning* strategy. As illustrated in Fig 8, once the sliding window completely moves to a new training task range, all blocks before the final global model block *M*_{*j*,*L*}_ for learning task *j* can be safely discarded because *M*_{*j*,*L*}_ contains all the necessary information for recording the learning process of task *j*. Furthermore, the model block *M*_{*j*,*L*}_ is an off-chain stored by the corresponding consensus committee responsible for block creation, and the on-chain storage contains only the block header. This strategy ensures that the block is stored in a distributed manner within a limited range of nodes, whereas the other nodes hold proof of content, thereby guaranteeing the immutability of the stored data. Therefore, the storage requirements are reduced without damaging system security.

## Theoretical analysis

In this section, FLCoin is analyzed from a theoretical perspective, including overhead and security.

### Communication overhead for consensus protocol operation

In this section, we analyze the additional communication overhead of our system for adopting blockchain in federated learning processing caused by the consensus protocol operation. In our system, we assume a total of *n* nodes, and each node *i* is either a participant or nonparticipant in the consensus protocol, denoted as *ϕ*_*i*_ ∈ {0, 1}. In addition, we use *ψ*_*i*_ ∈ {0, 1} to indicate whether node *i* is selected as the leader of the consensus protocol. In PBFT, all nodes must participate in the consensus process, with only one node selected as the leader in each round. Hence, $\sum_{i=1}^{n}\phi_{i}=n$ and $\sum_{i=1}^{n}\psi_{i}=1$. The number of messages that must be transmitted for PBFT consensus to reach consensus is *M*_*PBFT*_ = (*n* − 1) + *n*(*n* − 1) + *n*(*n* − 1) = 2*n*^2^ − *n* − 1.

For FLCoin, only the nodes that hold update shares in a sliding window with a fixed size *s* can participate in the consensus process, and multiple update shares can be held by a single node. Hence, committee size *c* satisfies *c* ≤ *s*. We assume *c* = *s*, which is the case that costs the most message exchanging, and one of the *s* nodes is selected as the leader; hence, $\sum_{i=1}^{n}\phi_{i}=s$ and $\sum_{i=1}^{n}\psi_{i}=1$. This protocol is divided into two consensus protocols: an optimistic fast protocol and a backup protocol. The former involves three phases and the latter involves five phases. The number of messages exchanged in the optimistic fast protocol is *M*_*fast*_ = 3(*s* − 1), and that in the backup protocol is $M_{backup}=\left(s-1\right)+\left(\frac{2s+1}{3}-1\right)+\left(s-1\right)+\left(\frac{2s+1}{3}-1\right)+\left(s-1\right)=\frac{13}{3}\left(s-1\right)$.

**Fig 9:**
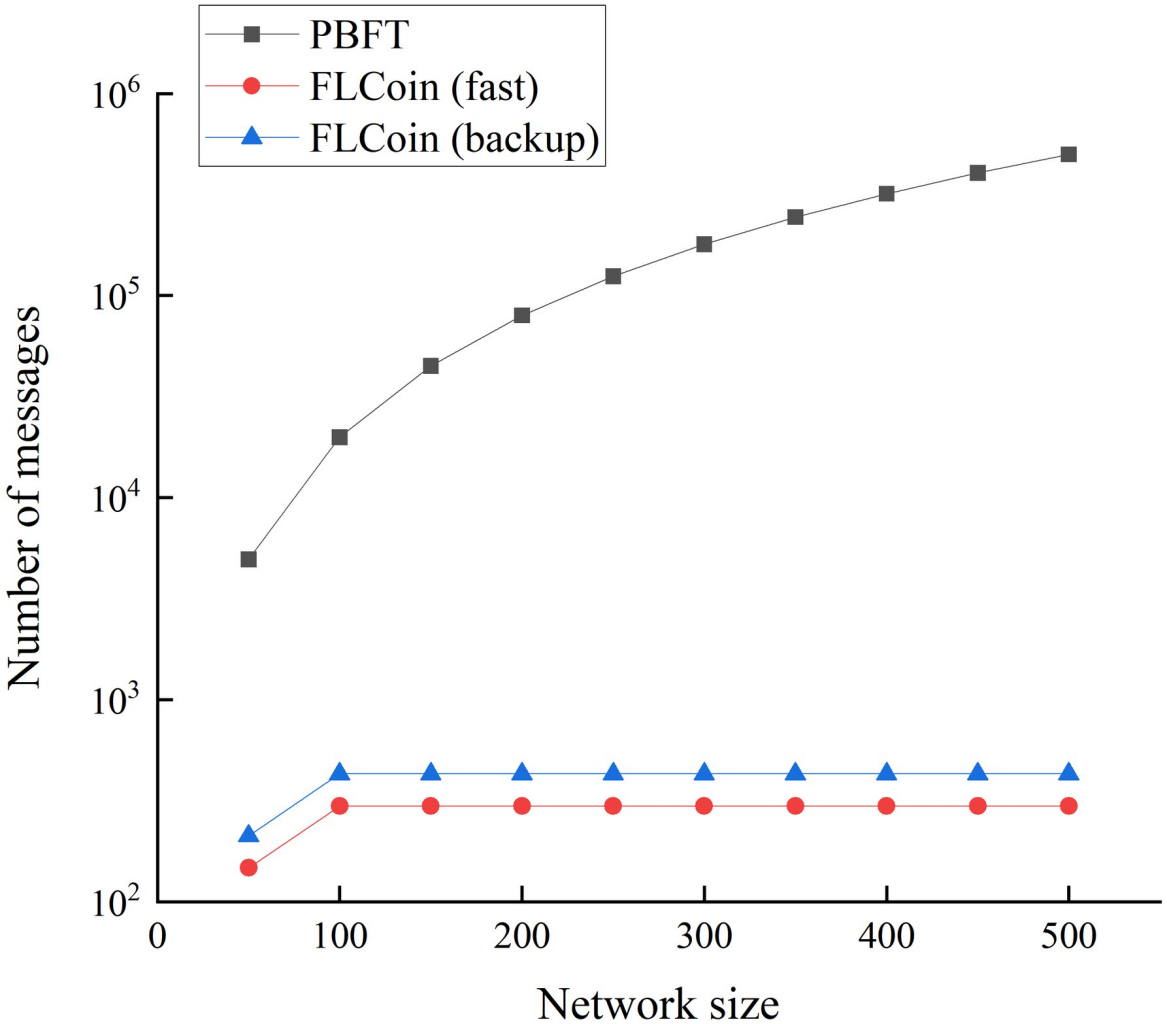
Communication overhead comparison based on different network sizes between PBFT and FLCoin protocols for sliding window size *s* = 100.

Fig 9 illustrates the impact of the network size *n* on the message transfer volume for both PBFT and FLCoin consensus protocols, considering a consensus sliding window size of *s* = 100 for FLCoin. As the number of nodes in the system increases, the number of messages transferred in PBFT increases exponentially, with *M*_*PBFT*_ exceeding 10^5^ when *n* = 300. By contrast, for FLCoin, the sliding window size *s* is fixed and irrelevant to *n*; thus, the number of messages transmitted in FLCoin remains stable regardless of the network size. Furthermore, the number of messages transmitted for both the optimistic fast protocol and backup protocol for FLCoin remained below 10^3^, demonstrating a substantial reduction of over 90% compared with the number of messages required for PBFT.

### Consensus committee security

For the successful execution of asynchronous BFT protocols, the proportion of adversaries must be less than 30%. Therefore, ensuring that the selected consensus committee has a high probability of having fewer than 30% malicious nodes is crucial. In our sliding window-based consensus committee selection approach, the nodes must participate in the learning task and consume resources to obtain the update block shares held in the current window. We assume that sliding window size *s* is equal to consensus committee size *c*. Therefore, the shares in the sliding window are held by different nodes. Consequently, the selection of the consensus committee can be expressed by a hypergeometric distribution, and the probability of selecting a successful committee is calculated as:

$$\begin{array}{c} P=\left[X\leq \frac{s}{3}\right]=\sum_{k=0}^{\frac{s}{3}}\frac{{(}_{i}^{K}){(}_{s-i}^{n-K})}{{(}_{s}^{n})} \end{array}$$ (5)

where *X* represents the number of malicious nodes included in a sliding window with the size of *s*, *n* is the total number of nodes in the system, and *K* represents the update blocks mined by the honest nodes.

**Fig 10:**
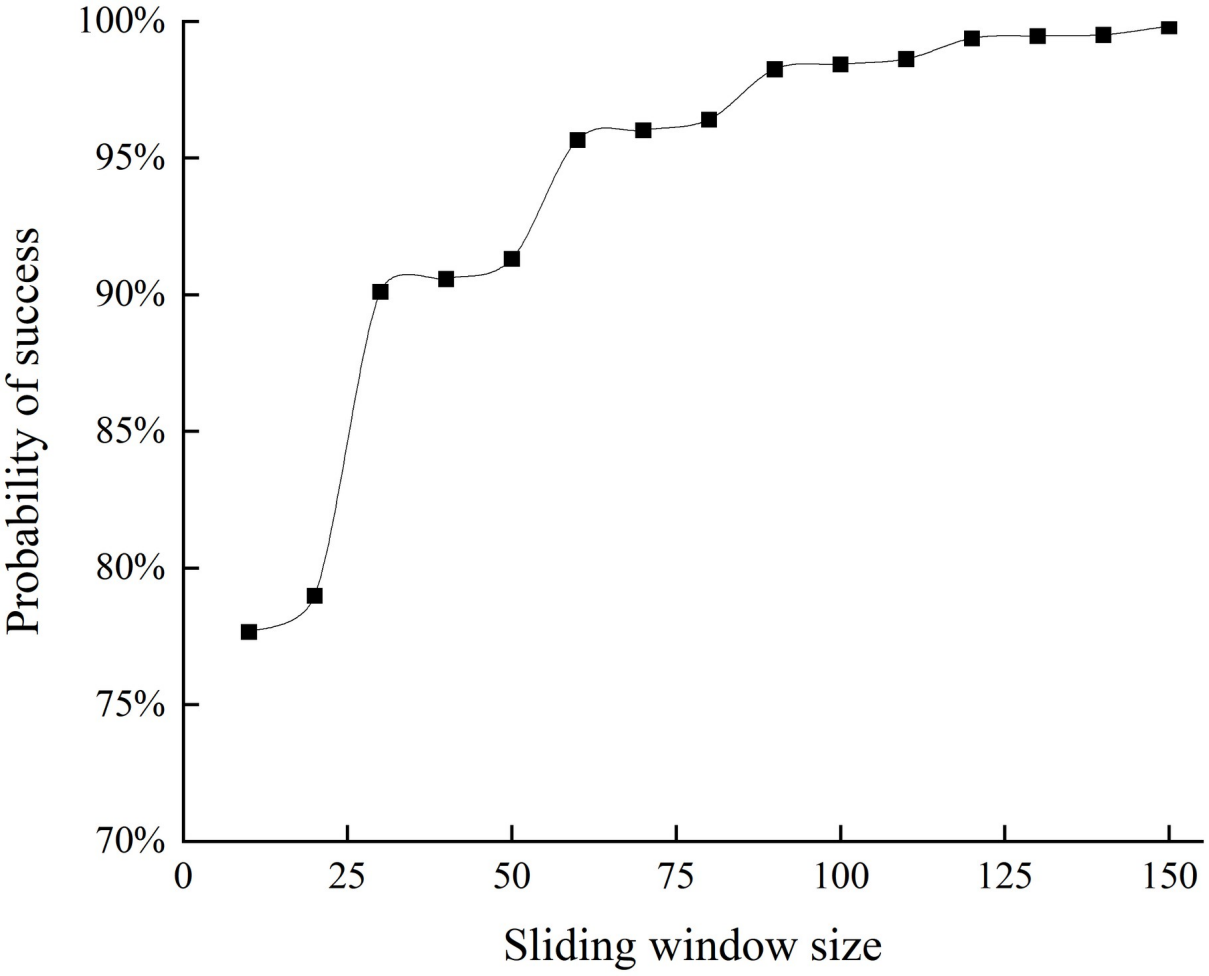
Success probability of committee election for network size *n* = 500.

Fig 10 shows the probability of selecting a secure committee based on different consensus committee sizes when 500 nodes are assumed in the system. The figure shows that the probability of selecting a successful committee increased as the sliding window size increased. The possibility of success is 90% when the sliding window size equals 30, and when the sliding window size is 150, the probability reaches 99.8%. However, the sliding window size must be balanced with the protocol communication overhead. To reduce communication overhead while ensuring the security of the consensus committee, we chose sliding window sizes of 50 and 100 for 500 nodes in the system. This choice resulted in a probability of selecting a secure consensus committee of 91.3% and 98.4%, respectively.

## Evaluation

In this section, we present a simulation-based evaluation of the proposed FLCoin protocol and analyze the results. Our primary objective in conducting this evaluation was to scrutinize the performance of the protocol across various conditions and ascertain the extent to which consensus delay affects the task-learning process.

### Experimental setup

To implement our consensus protocol, we utilized the Cothority framework and leveraged AWS EC2 virtual machines as our runtime environment. We equipped each instance with 4vCPUs and 16GB of RAM to simulate edge computing conditions. The additional latency in adopting the blockchain in the federated learning process is mostly caused by the consensus protocol. Therefore, we evaluated the performance of the consensus protocol of the system. We measured latency, which reflects the time required to reach a consensus on the newly generated blocks. To demonstrate the efficiency of our protocol, we compared its performance with those of PBFT and ByzCoin. In addition, we conducted experiments to demonstrate how our protocol can effectively reduce the impact of consensus processing on the learning operation. To this end, we compared the performance of our protocol with that of the classic PBFT protocol as a consensus method for blockchain-based federated learning system. Furthermore, we assessed the training efficiency of the proposed architecture by conducting a comparative analysis with Biscotti, a representative approach that utilizes the same training dataset [23]. The parameters used in the experiments are shown in Table 2. All the experiments were conducted under the same constraints using the same testbed. In this section, we focus primarily on evaluating the effectiveness and efficiency of the proposed architecture. The security of our consensus protocol was confirmed through theoretical analysis, as described in the previous section. In addition, it is important to note that updates uploaded by malicious nodes can be filtered by a consensus committee. Therefore, for our experiments, we assumed that there were no malicious participants in the system.

**Table 2:** Parameter settings of experiments.

Parameter name	Value
Training model	LeNet-5
Dataset	MNIST
Training dataset size	60000
Sample size of each training node	600
Batch size	200
Learning rate	0.01

### Consensus protocol latency

We evaluated the performance of the protocol by measuring the latency, which is the time required to add a new block to the blockchain. The key factors influencing this metric are the block size and number of nodes involved in the consensus process. In the first experiment, 500 nodes were considered in the system. We set the sliding window size *s* to 50 and 100 to balance the trade-off between communication overhead and consensus committee security. Our consensus protocol incorporates optimistic fast and backup protocols, so we ran both protocols separately for block sizes ranging from 0.5MB to 5MB to assess the latency.

**Fig 11:**
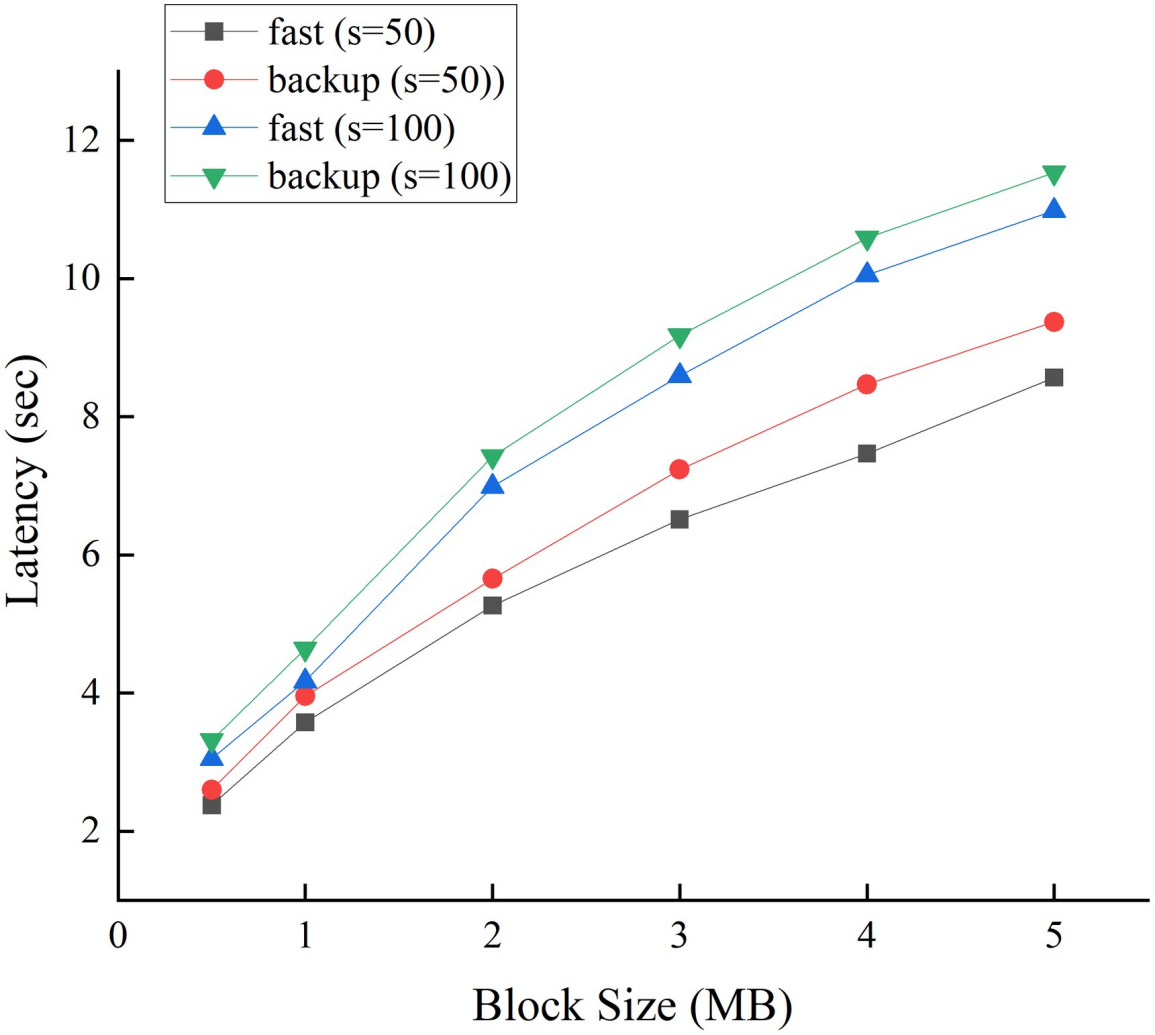
Latency of fast and backup protocols for network size *n* = 500, sliding window size *s* = 50, and *s* = 100.

Fig 11 illustrates that the latency of both the fast and backup protocols increase with larger block sizes. When the sliding window size *s* was set to 50, which offers a 91.3% chance of selecting a secure committee. In this case, the consensus latencies of both protocols remain below 4 seconds for a block size of 1M, which is commonly used in most blockchains. Even for the large block size of 2M, the consensus latencies of both protocols remained within a reasonable range, i.e., less than 6 s. When *s* was increased to 100, providing a 98.4% probability of selecting a secure committee, the latency of both protocol modes remained below 5 s, and the latency increased to approximately 7 s for a block size of 2M. We opted for a linear communication mode instead of the broadcast communication mode used in PBFT. Consequently, the latency did not increase significantly despite the two additional communication rounds required by the backup protocol compared with the fast protocol.

**Fig 12:**
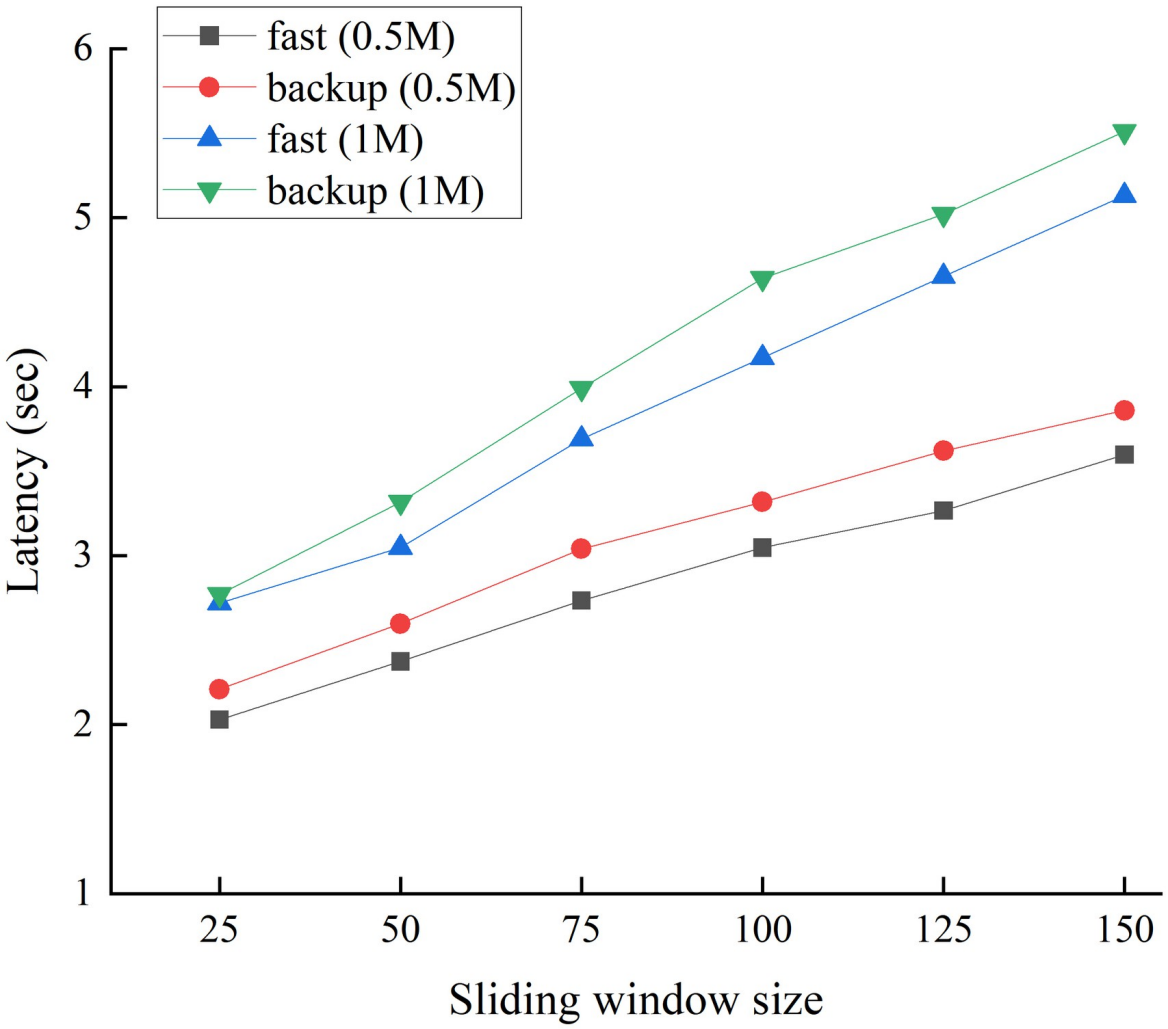
Latency of fast and backup protocols for network size *n* = 500 with blocks of 0.5M and 1M.

To evaluate the impact of the number of nodes involved in the consensus process on the protocol latency, we conducted another experiment in which we gradually increased the sliding window size *s* from 25 to 150. We repeated the experiment using two block sizes for the fast and backup protocols. Fig 12 shows the consensus latency of both protocols for block sizes of 0.5 and 1 MB. As in previous experiments, the protocol latency increased proportionally with the block size. Additionally, as the number of consensus committee members increased, the latency increased linearly. However, because of the low communication complexity of our proposed protocol, even when *s* was increased to 150, the latency of both protocols remained below 6 s to reach a consensus on a block of 1M.

**Fig 13:**
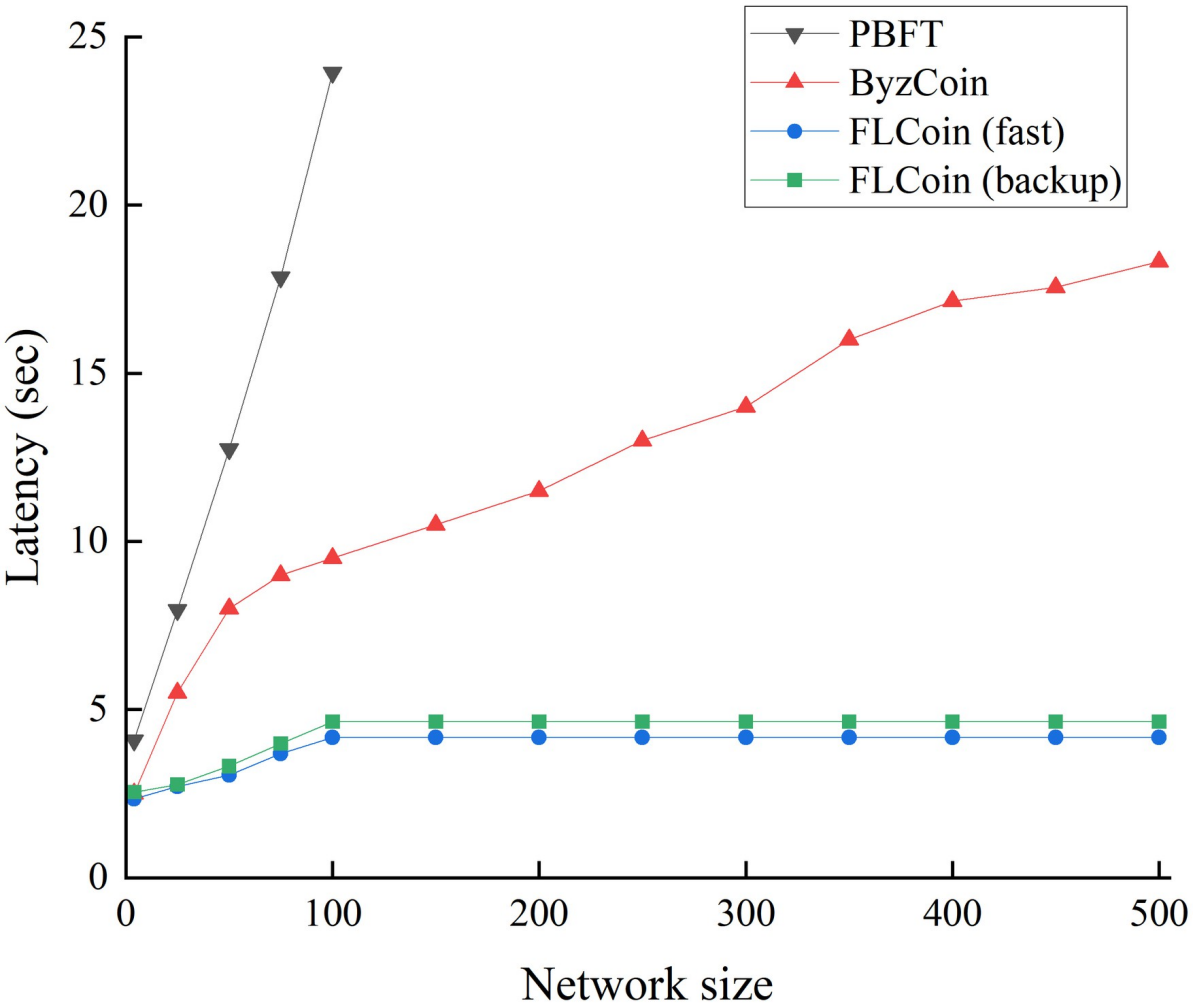
Performance comparison with different network sizes.

To evaluate the efficiency of the proposed FLCoin method, we compared it to the PBFT and ByzCoin protocols. We gradually increased the total number of nodes *n* included in the system from 4 to 500 while fixing the block size at 1 M and compare the consensus latency of the protocols. To ensure the security of the consensus committee in FLCoin, we set the sliding window size *s* as 100. When *n* ≤ 100, *s* = *n*, and when *n* > 100, *s* = 100. The results in Fig 13 show the latency required for different protocols to reach a consensus under varying network sizes. As expected, in small-scale network environments, PBFT, ByzCoin, and the proposed protocols, including fast and backup protocols, can maintain low-latency high-speed operation. However, owing to the high communication complexity *O*(*n*^2^), the consensus latency of PBFT is already close to 25 s when the total number of network nodes reaches 100. In comparison, ByzCoin achieves a better performance by using collective signing and more efficient communication patterns. The consensus delay of our protocol maintains a small linear growth with an increase in the network size. When the total number of system nodes exceeded 100, the number of nodes participating in the consensus processing adopted a fixed value. At this point, the consensus latency stabilizes and does not vary with network size. The latency of our proposed fast and backup protocols can be maintained at less than 5 s during an increase in network size, ensuring high efficiency. Furthermore, our security analysis showed that the chosen sliding window size of *s* = 100 is sufficient to maintain the security of a system containing 500 nodes.

### Latency of learning and consensus processing

**Fig 14:**
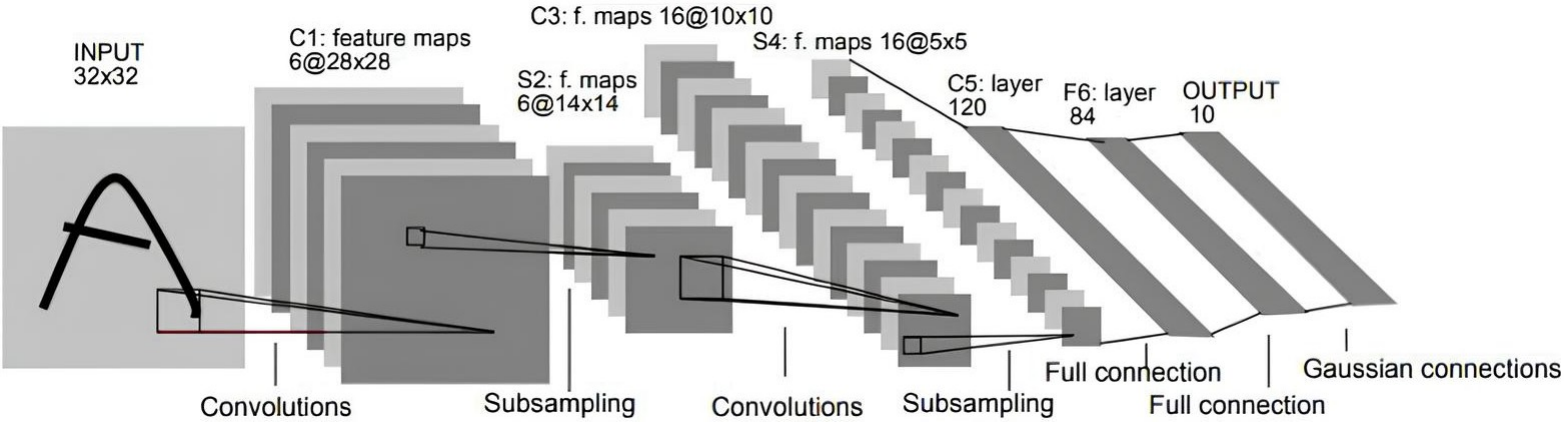
LeNet-5 architecture.

**Fig 15:**
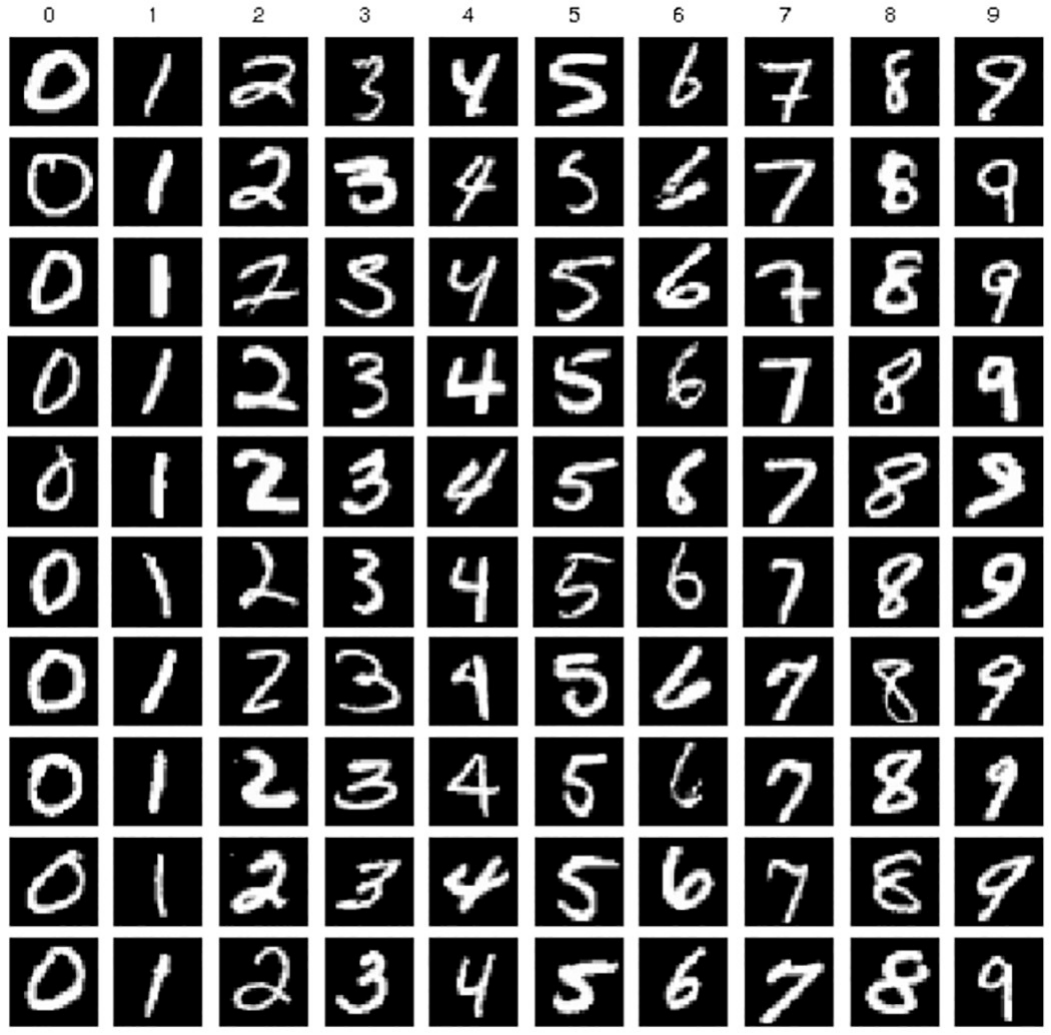
Example images from the MNIST dataset.

In a blockchain-based federated learning system, the central node is replaced by a blockchain for global model aggregation, which introduces additional latency owing to consensus processing. Our proposed framework uses a consensus committee to replace the central node and executes an optimized BFT protocol to reach consensus on a newly generated model block. To analyze the impact of consensus latency on the learning process, we trained the LeNet-5 convolutional neural network (CNN), which is a simple image classification model with approximately 0.6M parameters [24]. As shown in Fig 14, the LeNet-5 network comprises seven layers: three convolutional layers, two pooling layers, and two fully connected layers. Model training was based on the MNIST dataset, which consisted of 60,000 training images and 10,000 test images [25]. As illustrated in Fig 15, each image is a grayscale image with dimensions of 28x28 pixels, represented as matrices, where each pixel value ranges from 0 to 255 to indicate grayscale intensity. Corresponding labels are provided for each image in the dataset, denoting the digit it represents to facilitate model training and evaluation.

**Fig 16:**
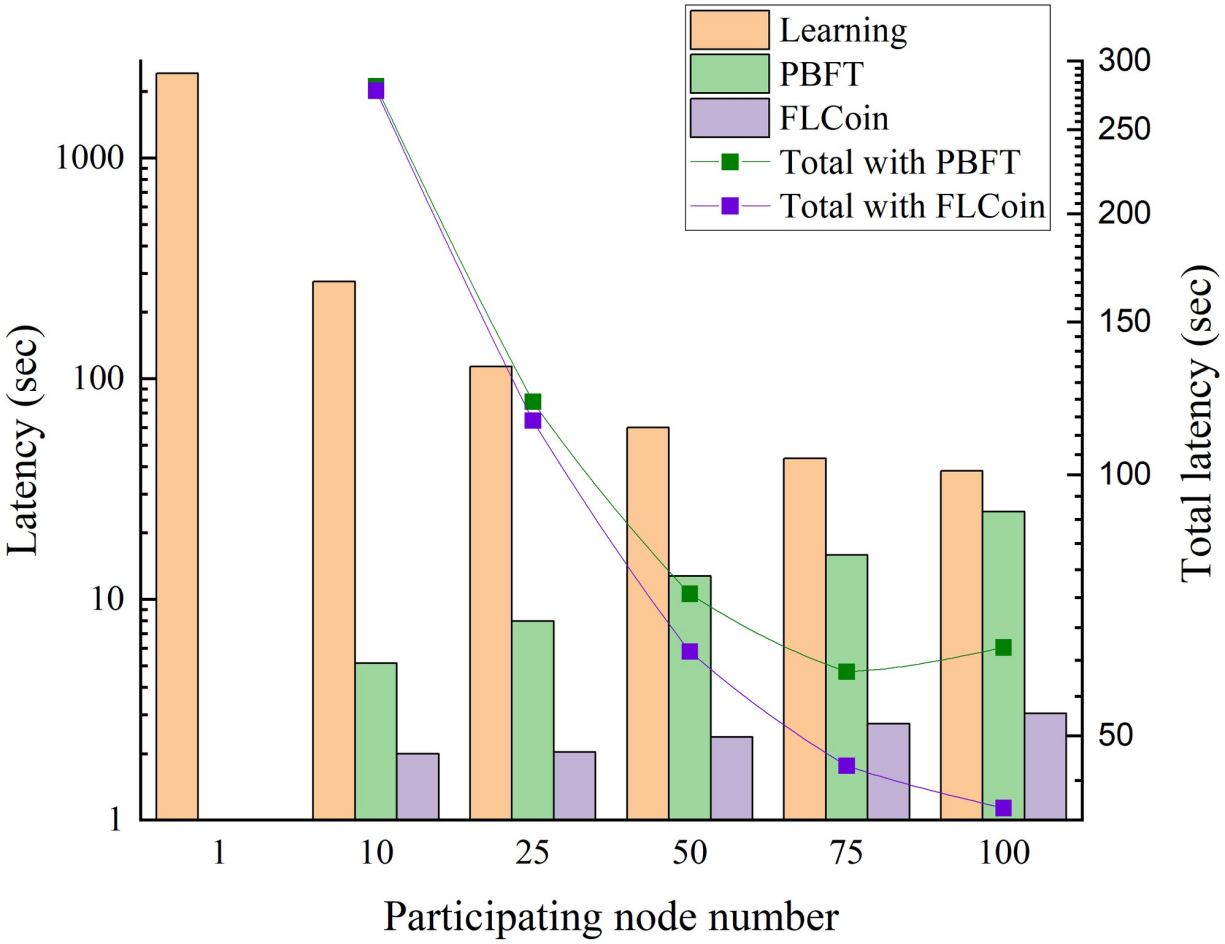
Latency comparison of different numbers of participating nodes.

Each training round had 40 training epochs, and the number of training nodes increased from 1 to 100. We compared the consensus latency of PBFT and our proposed FLCoin method with the learning time, as illustrated in Fig 16. As expected, the learning time decreased with an increase in the number of training nodes, resulting in a gradual decrease in the total latency based on both PBFT and FLCoin consensus protocols. However, the consensus latency increases with the number of participating nodes. Using PBFT as the consensus protocol led to a significant increase in latency during a node increase. When the system has 100 nodes, the latency is nearly the same as the learning time and the total latency starts to increase, which is a bottleneck for the scalability of the system. By contrast, using our proposed FLCoin method, the increase in latency was much smaller, resulting in a negligible impact on the learning process. We also compared the time required to achieve an accuracy of 97.3%. The comparison results between the training based on PBFT and FLCoin are listed in Table 3.

**Table 3:** Time cost comparison of training with PBFT and FLCoin.

Number of training nodes	Training with PBFT		Training with FLCoin	
	Consensus latency (s)	Training time (s)	Consensus latency (s)	Training time (s)
25	7.96	121.46	2.03	115.53
50	12.74	72.92	2.37	62.55
75	15.85	59.31	2.73	46.14
100	25.11	63.24	3.05	41.29

**Fig 17:**
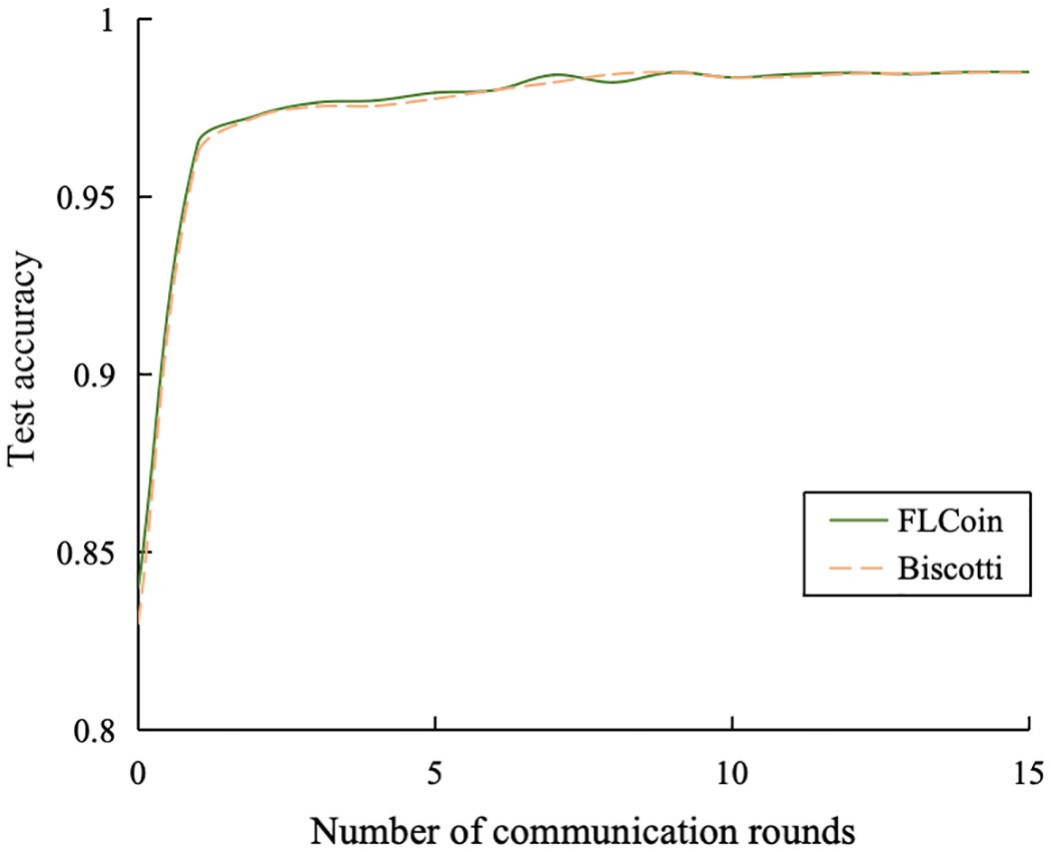
Comparison of test accuracy for Biscotti and FLCoin.

**Fig 18:**
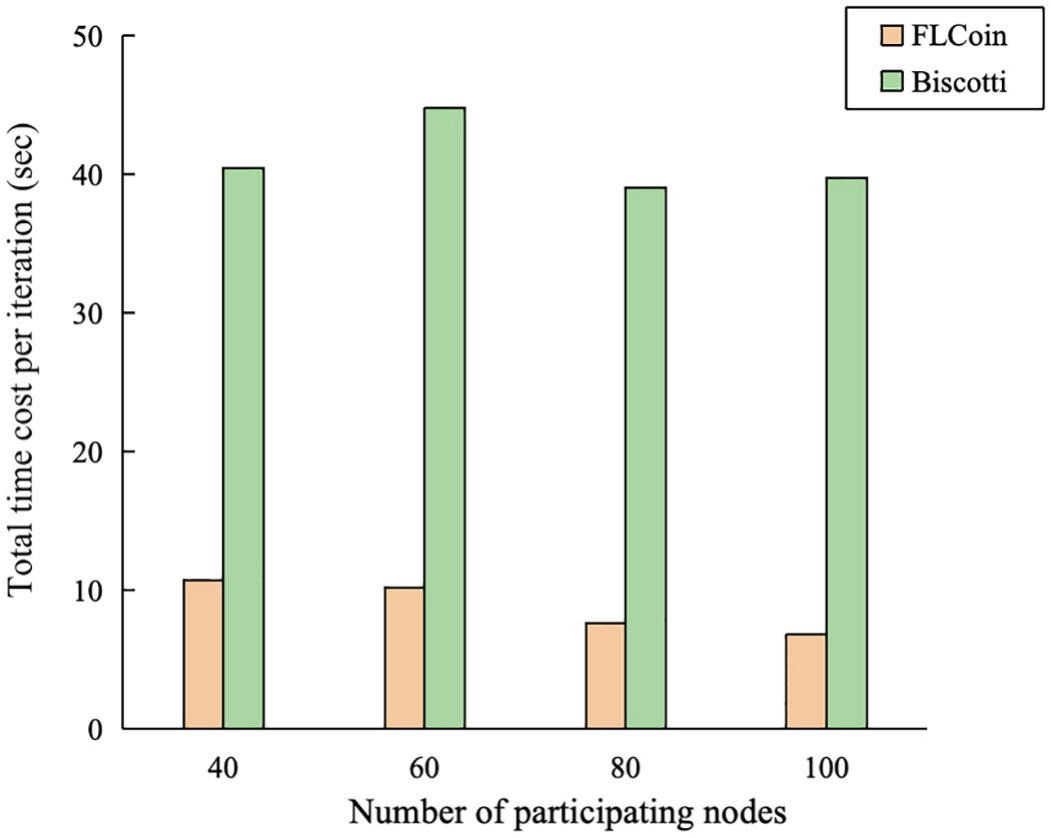
Per training iteration times with varying numbers of participating nodes.

FLCoin demonstrates superior efficiency compared to Biscotti, a prominent FL approach based on blockchain systems utilizing Proof-of-Federation consensus protocol [23]. Similar to our methodology, Biscotti framework underwent an evaluation study using the MNIST dataset. The test accuracies of the two systems are presented in Fig 17. It can be observed that the convergence speeds of the two systems are very close. We further compared the times required by the two systems for each training iteration, and the results are plotted in Fig 18. With 100 nodes engaged in the model training, Biscotti required approximately 40 s per iteration. By contrast, our proposed architecture completes each training iteration in less than 7 s.

## Related work

In recent years, privacy has become an increasingly important concern. Khairy et al. conducted a comprehensive study on Facebook usage in Egypt, focusing on aspects such as privacy, reporting systems, and cyberbullying incidents [26]. Lotfy et al. experimentally investigated privacy concerns related to public Wi-Fi usage [27]. There is no doubt that blockchain is one of the most inspiring technologies, which can solve the above-mentioned issues. El Koshiry et al. demonstrated the potential benefits of integrating blockchain into the education system, highlighting improvements in the efficiency, security, and credibility of educational processes [28]. As Bitcoin has become widely known, Proof-of-Work (PoW), as the consensus protocol of Bitcoin’s underlying blockchain and the earliest permissionless blockchain consensus protocol, has also been widely used. By contrast, Practical Byzantine Fault Tolerance (PBFT), as an early proposed consensus protocol for distributed systems, has been favored by private blockchains, providing another solution for blockchain consensus. In the context of blockchain-based federated learning (FL) structures, both PoW and PBFT are commonly utilized. For instance, FLChain adopts PBFT or PoW as its underlying consensus protocols [11]. Similarly, other studies proposed blockchain-based FL schemes employing PoW as the consensus protocol [29, 30]. In addition, blockchain-based FL structures were tailored to address specific scenarios. For instance, Bouachir et al. introduced FederatedGrids as an energy-sharing platform by employing a PoW-based Ethereum blockchain along with federated learning [31]. Islam et al. presented an FL method that leverages a PoW-based blockchain to aggregate data sourced from IoT devices [32]. Baucas et al. devised a Fog-IoT platform dedicated to predictive healthcare. This platform utilized federated learning and a PBFT-based private blockchain [33]. However, PoW is not an optimal choice as a consensus protocol in a federated learning environment because of its long latency. However, PBFT is not suitable for large-scale networks. PoFL is the first consensus protocol that relies directly on federated learning [34]. By replacing PoW with federated learning, the energy wasted in solving meaningless puzzles in PoW is reinvested into federated learning. However, global model aggregation relies on the mining pool manager, which can cause a centralization problem. Recently, Lian et al. proposed a blockchain-based personalized federated learning for the Internet of Medical Things (IoMT) [35]. They utilized Proof of Stake (PoS) as a consensus protocol to replace PoW, which avoids huge resource consumption. However, as a consensus protocol, PoS faces other vulnerabilities, such as the nothing-at-stake problem, in which validators have nothing to lose by supporting multiple conflicting chains. Li et al. proposed BFLC, which provides a committee-based option for a blockchain-based federated learning framework [36]. By employing a consensus committee, the efficiency of the consensus protocol and scalability of the system can be improved. However, BFLC requires all committee members to verify all local learning updates with their dataset, which requires additional resources.

Outside federated learning settings, committee optimized PBFT protocols have recently emerged to improve the scalability of the classic PBFT protocol. One such protocol is DBFT, which selects a consensus committee to validate blocks based on the PBFT rules [37]. Representatives were elected by voting, and the consensus protocol was run to generate blocks. DBFT improves the scalability and reduces the latency of PBFT; however, its average latency of 15 s is still too high for low-latency edge computing networks. ByzCoin combines the PoW and PBFT protocols using the underlying PoW blockchain to select and update a consensus committee via a sliding window [19]. However, this protocol still wastes computing power resources owing to its dependence on PoW. G-PBFT avoids this problem by selecting a consensus committee in a location-based manner [38]. Nodes staying in the same location for over 72 h were eligible to be elected as committee members, and malicious nodes were removed. This approach eliminates the waste of computation resources, but does not impose any penalties on malicious nodes, which can undermine the security of the system.

Another approach for improving the scalability of PBFT is to use a two-layer structure. DR-BFT uses a two-layer blockchain with private chains as the bottom layer, where private chain leaders run the consensus protocol as a consensus committee of the top layer chain [39]. By contrast, DP-Hybrid runs the PBFT protocol in the bottom groups to generate blocks, whereas the PoW protocol at the top layer selects a unique block from the resulting blocks [40]. By contrast, multi-PBFT achieves a top-down consensus by first running the PBFT protocol at the top layer. The consensus committee members at the top layer serve as the primary nodes of the groups at the next layer to deliver consensus information to the groups [41]. Although these multilayer structures can improve PBFT scalability, they still face challenges such as large message delivery and high latency.

Moreover, shard-structured blockchains, such as Elastico, Omniledger, and Rapidchain improve scalability by independently handling the consensus of each shard [42–44]. Inspired by these designs, the next step in our research is to extend FLCoin into a framework that enables the parallel training of different learning tasks simultaneously on different sub-chains, further enhancing its scalability and efficiency.

## Limitations and future work

Currently, our experiments to evaluate the effectiveness of model training within our proposed architecture operate under the assumption that there are no malicious nodes without considering the potential impacts of such entities. However, it is important to acknowledge that malicious nodes can pose threats to system robustness and global model accuracy, potentially through poison attacks, although this is extremely unlikely. In our future work, we aim to analyze the performance of our architecture in the presence of malicious nodes to assess their impact on system robustness.

In addition, our current approach faces challenges in handling the training of large-scale deep learning models, with millions of parameters being transported among training servers, which is also a common issue in FL schemes. To address this problem, we plan to explore the integration of model partitioning and compression technologies into our methodology. This integration is expected to enhance the ability of the proposed system to efficiently train large-scale models.

## Conclusion

In this study, we proposed the FLCoin architecture tailored to IoT-based edge computing scenarios. By utilizing federated learning, the architecture can select a trustworthy consensus committee and improve the scalability and efficiency of edge computing systems. Moreover, the optimistic, fast, and backup protocols of the consensus mechanism can further reduce latency while ensuring security. Resultantly, our approach provides an efficient and scalable solution for IoT edge networks by integrating blockchain and federated learning. An evaluation revealed that the proposed method demonstrated excellent performance. Compared to a PBFT blockchain-based FL system, our approach achieves 90% gain in communication overhead and a 35% reduction in training time cost. With its high scalability and low latency, FLCoin proved to be a superior integration architecture of blockchain and federated learning for edge computing scenarios. In future work, we plan to extend the FLCoin architecture to support the simultaneous training of learning tasks on different sub-chains, further enhancing scalability and flexibility.
